# HNdb: an integrated database of gene and protein information on head and neck squamous cell carcinoma

**DOI:** 10.1093/database/baw026

**Published:** 2016-03-24

**Authors:** Tiago Henrique, Nelson José Freitas da Silveira, Arthur Henrique Cunha Volpato, Mayra Mataruco Mioto, Ana Carolina Buzzo Stefanini, Adil Bachir Fares, João Gustavo da Silva Castro Andrade, Carolina Masson, Rossana Verónica Mendoza López, Fabio Daumas Nunes, Luis Paulo Kowalski, Patricia Severino, Eloiza Helena Tajara

**Affiliations:** ^1^Department of Molecular Biology, School of Medicine of São José do Rio Preto, SP, Brazil Av Brigadeiro Faria Lima n° 5416 Vila Sao Pedro 15090-000 - São José do Rio Preto, SP - Brazil; ^2^Institute of Exact Science, Federal University of Alfenas, MG, Brazil, Rua Gabriel Monteiro da Silva, 700 Centro 37130-000 - Alfenas, MG - Brazil; ^3^Department of Dermatological, Infectious, and Parasitic Diseases, School of Medicine of São José do Rio Preto, SP, Brazil Av Brigadeiro Faria Lima n° 5416 Vila Sao Pedro 15090-000 - São José do Rio Preto, SP - Brazil; ^4^Department of Genetics and Evolutionary Biology, Institute of Biosciences, University of São Paulo, SP, Brazil R. do Matão Butantã 05508-090 - São Paulo, SP, Brazil; ^5^State of São Paulo Cancer Institute – ICESP, SP, Brazil Av. Dr. Arnaldo, 251 Pacaembu 01246-000 - São Paulo, SP - Brazil; ^6^Department of Stomatology School of Dentistry, University of São Paulo, SP, Brazil Avenida Professor Lineu Prestes, 2227 Butantã 05508-000 - São Paulo, SP - Brazil; ^7^Department of Head and Neck Surgery and Otorhinolaryngology, Cancer Hospital A.C. Camargo, SP, Brazil Rua Prof Antonio Prudente, 211 Liberdade 01509-010 - São Paulo, SP - Brazil and; ^8^Albert Einstein Research and Education Institute, Hospital Israelita Albert Einstein, SP, Brazil Av. Albert Einstein, 627 Morumbi 05652-000 - São Paulo, SP - Brazil

## Abstract

The total amount of scientific literature has grown rapidly in recent years. Specifically, there are several million citations in the field of cancer. This makes it difficult, if not impossible, to manually retrieve relevant information on the mechanisms that govern tumor behavior or the neoplastic process. Furthermore, cancer is a complex disease or, more accurately, a set of diseases. The heterogeneity that permeates many tumors is particularly evident in head and neck (HN) cancer, one of the most common types of cancer worldwide. In this study, we present HNdb, a free database that aims to provide a unified and comprehensive resource of information on genes and proteins involved in HN squamous cell carcinoma, covering data on genomics, transcriptomics, proteomics, literature citations and also cross-references of external databases. Different literature searches of MEDLINE abstracts were performed using specific Medical Subject Headings (MeSH terms) for oral, oropharyngeal, hypopharyngeal and laryngeal squamous cell carcinomas. A curated gene-to-publication assignment yielded a total of 1370 genes related to HN cancer. The diversity of results allowed identifying novel and mostly unexplored gene associations, revealing*,* for example, that processes linked to response to steroid hormone stimulus are significantly enriched in genes related to HN carcinomas. Thus, our database expands the possibilities for gene networks investigation, providing potential hypothesis to be tested.

**Database URL:** http://www.gencapo.famerp.br/hndb

## Introduction

The high-throughput ‘omics’ technologies (genomics, transcriptomics, proteomics and metabolomics) and advanced computational tools have led to a more thorough understanding of the neoplastic process as well as to the identification of potential biomarkers for cancer diagnosis and prognosis. These high-throughput technologies accumulate scientific data on an unprecedented scale. However, these data are dispersed between several databases, including, *inter alia*, The Cancer Genome Atlas (TCGA) (1) Gene Expression Omnibus (GEO) (2), ONCOMINE (3), the Human Protein Atlas (4) and the Human Metabolome Database (HMDB) (5–7). This decentralized structure poses substantial problems when attempting to draw conclusions or formulate new hypotheses.

PubMed (8), a freely available database developed and maintained by the US National Library of Medicine, is one of the most important web-based search tools for biomedical information retrieval. Currently, PubMed has over 3 million citations on cancer. Thus, it is extremely difficult to manually retrieve all relevant data, even after splitting the subarea of interest or using specific queries. In addition, literature searches on cancer are hampered by the fact that cancer is a complex disease. Cancer and cancer subtypes more closely resemble a set of diseases, each disease with different features and unknowns. Head and neck (HN) cancer is the sixth most common type of cancer worldwide, with about 600 000 new cases in 2012 (9) and a remarkable example of heterogeneous malignancy.

Similar to what is observed in many types of neoplasms, the challenge in searching the literature on HN cancer is particularly difficult due to its diversity, which involves diversity in histological type, anatomical location and primary risk factors. For instance, the anatomical sites affected by the disease and the primary risk factors can be used to divide head and neck squamous cell carcinomas (HNSCC) into at least three classes. Two of these classes involve human papillomavirus (HPV)-positive disease (mostly oropharyngeal with a favorable prognosis) and HPV-negative disease (with less favorable prognosis and a different molecular profile) (10). HPV-positive tumors are primarily wild-type *TP53*, whereas HPV-negative tumors present mutated *TP53* and show high chromosome instability (10, 11), which may sustain advantageous metabolic pathways, aid in escaping the inhibitory effects of suppressor signals (12) or promote oncogenic effects (13). A third class of HNSCC consists of nasopharyngeal tumors in which distinct etiological factors are known, including Epstein–Barr virus (EBV) infection (14).

Lymph node status and tumor size remain the most powerful prognostic factors for HNSCC. However, survival is frequently low. Only 40–50% of patients remain alive 5 years after diagnosis (10, 15). This is likely because tumors in early stages frequently present few symptoms leading to a delay in diagnosis. Furthermore, therapy effectiveness is highly variable, even in early lesions or histologically similar cases.

The HNSCC molecular progression model suggests that some genetic alterations are present in benign hyperplasia, for instance the inactivation of the *CDKN2A* gene. According to this model, the clinical progression to dysplasia, *in situ* carcinoma, and, finally, invasive carcinoma is supported by the increased accumulation of molecular alterations (16). *TP53* mutations, *CCND1* gene amplification, *EGFR* activation/*PTEN* inactivation, and the deletion of different genome segments are some examples of the genetic alterations related to HNSCC progression, as stated by Leemans and collaborators (10). Such alterations promote the neoplastic phenotype defined by Hanahan and Weinberg (17), including increased cell proliferation, insensitivity to growth suppression factors, apoptosis resistance, sustained angiogenesis, energy metabolism alterations, immune attack avoidance and the acquisition of invasion and metastasis capability.

Investigations into HNSCC emphasize the importance of identifying the mechanisms and the molecular changes triggered during the malignant transformation that culminates in the neoplastic phenotype. New data on potential markers may shed light on tumor biology and, consequently, lead to the development of novel drugs. Literature mining is a fundamental starting point for this discovery process, but the recent exponential growth in biological data is well beyond the limit of a complete manual search in most cases. In turn, automated literature mining can help to find disease-related biomarkers and their interrelationships, and extract hidden information with tools able to efficiently target valuable research questions and generate testable hypothesis. During this process, the articles of interest are retrieved, the biological entities are identified in texts, and specific information, particularly relationships between biological entities, is extracted.

One of the challenges in automated approaches is the exact identification of genes, proteins or diseases since they may be referred to by different names, share names and symbols, or even be described by nonstandard nomenclature in literature and databases (18). Another challenge is to identify consistent descriptions of gene products and their associated features, and supporting evidence for inferring such associations. To overcome these limitations, text-mining applications have incorporated tools to recognize specific keywords and to capture relevant sentences and ontologies. For example, relationships may be extracted investigating entities that co-occur in the same report, title, abstract or even a sentence, or by the so-called natural language processing (NLP) methods. NLP methods are based on the structure of sentences and on how the biological data is mentioned (19). However, this approach has advantages and limitations, since it may give rise to erroneous relationships depending on used parameters (20).

The controlled vocabularies of the Gene Ontology (GO) (21) project enable coupling of gene products to their associated biological processes, cellular components and molecular functions (22). However, the automatic identification of GO-literature association is less accurate than manual curation methods, such as the one using Medical Subject Headings (MeSH) (23) for indexing PubMed articles, a process performed by trained experts that potentially generates few false positive assignments. In addition, MeSH-literature associations may be linked to genes or diseases, facilitating the identification of previously unrevealed relationships between entities, such as protein–protein, drug–effect and protein–disease (24, 25).

In this work, we developed an in-house methodology to conduct literature mining aiming to identify genes and gene products related with various aspects of HNSCC. A database (HNdb) was established for unifying the information on these genes and proteins, covering data on genomics, transcriptomics, proteomics, literature citations, and also cross-references of external databases. The information was wrapped up in a friendly web interface, which provides easy and rapid access to the HNSCC-related genes and to a vast number of biological data resources*.* The interfaces aims to facilitate the selection of candidates for validation assays and the identification of potential new markers, as exemplified in this study.

## Methods

### Data collection and literature mining

**Figure 1.**
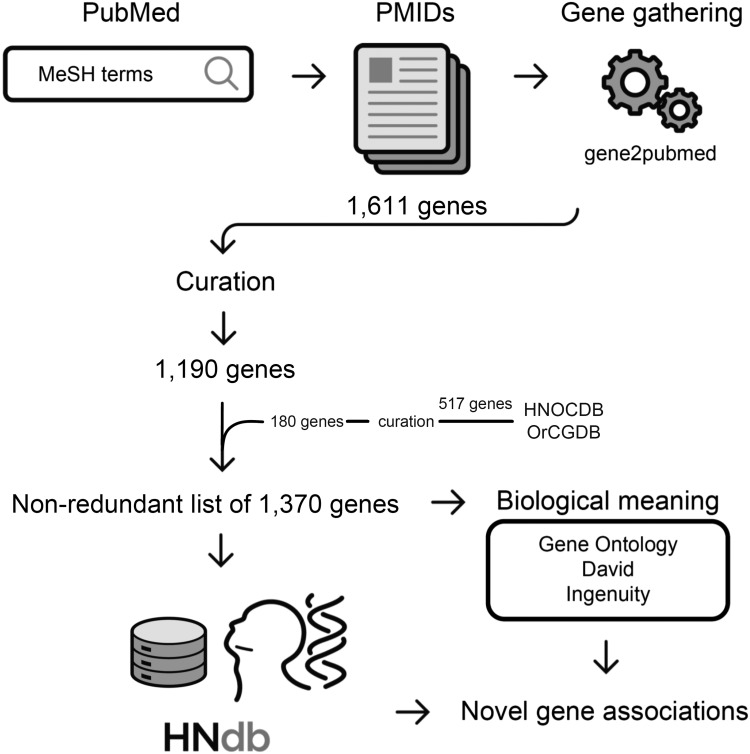
Flowchart of the method for gathering genes related to HNSCC. Identification of studies on HNSCC in PubMed using MeSH terms of interest, retrieval of PMIDs, gene-to-publication assignment via gene2pubmed association file, selection of genes in HNOCDB and OrCGDB databases, manual curation to confirm a positive involvement of PMIDs in HNSCC subsites of interest, retrieval of a nonredundant list of 1370 genes related to HNSCC, access to biological meanings, identification of novel gene associations.

The workflow of our literature mining consisted of two initial automated stages and a separate manual step. In stage I, the studies were retrieved from PubMed database using a combination of MeSH terms and Boolean operators. Three literature searches based on different MeSH terms were run on 29 June 2015. In stage II, the articles selected in stage I were associated with genes using the gene2pubmed association file (26), which contains the gene identifiers (gene IDs) and the respective PubMed article identifiers (PMIDs). For this association, only human genes were accepted. The PMIDs thereby obtained were downloaded via PubMed and compiled, and publications assigned to MeSH terms for HN neoplasms were manually curated by two independent investigators. The details on the MeSH terms and on the literature search strategy are presented in Supplementary File 1 and an overview of the workflow is provided in Figure 1.

Considering that our automated strategy may have missed relevant articles and genes, the only two databases, to our knowledge, that also focus on HNSCC were searched: the Head and Neck and Oral Cancer Database (HNOCDB) and Oral Cancer Gene Database (OrCGDB) (27, 28). PMIDs/genes not detected by our approach but selected by these databases were included in our list after manual curation to confirm a positive involvement with the HNSCC sites of interest. Precision (specificity) and recall (sensitivity) values were calculated, respectively, as the proportion of genes relevant from our search, and as the proportion of relevant genes that were retrieved [Precision = genes retrieved and relevant/total genes retrieved; Recall = genes retrieved and relevant/total genes relevant in collection]. To overcome the difficulty of predicting the total number of genes in PubMed that are relevant for our search, we used HNOCDB and OrCGDB data on the same query.

To establish a gene-to-HNSCC association, contingency tables were constructed using the curated set of articles addressing genes in HNSCC, and PMIDs and genes from all other neoplasms. Fisher’s exact test was performed to evaluate association and *P* <  0.05 were considered statistically significant. The analyses were performed using SAS® 9.3 (SAS Institute Inc., Cary, NC, USA) for Windows. Genes were then ranked according to their level of association with HNSCC—from the most relevant to the less relevant defined by the number of publications addressing the gene in HNSCC—by a hypergeometric test (29) performed using the Stirling's approximation to high-factorial values (30). The method calculates the probability of *k* or query-relevant publications for a gene A by chance, being *S* the score for gene A, *m* the publications in the gene2pubmed association file, *n* the number of publications retrieved for the query and present in the gene2pubmed association file, *j* the number of publications that involve gene A, and *k* the number of query-relevant publications that involves the gene A. The formula (ln= natural logarithm) is:

$$\begin{array}{l} S_{\text{A}}=\text{In}\left(f\left(m,n,j,k\right)\right)\\ f\left(m,n,j,k\right)=\sum_{i=k}^{\min\;\left(n,j\right)}\frac{\left(\begin{array}{c} m-j\\ n-i \end{array}\right)\left(\begin{array}{c} j\\ i \end{array}\right)}{\left(\begin{array}{c} m\\ n \end{array}\right)} \end{array}$$

Due to the importance of identifying prognostic signatures for HNSCC as well as markers associated with disease progression, an independent search was performed using MeSH and non-MeSH terms related to ‘Metastasis’ and ‘Prognosis/Outcome’ against abstracts and titles of the manually curated PMID set of articles (Supplementary File 1).

The data collection workflow will be routinely updated twice per year to incorporate new PMIDs and genes.

### Database frameworks and web interface

To integrate potential biomarkers involved in HNSCC with data from the available literature, we constructed a MySQL relational database system implemented in an Apache server using the Linux operating system. The web platform interface was developed using the JavaScript programming language, HTML and PHP at the front end and the back end supported by PHP and PERL programming languages. The platform provides users with the ability to search for and download information on the genes and proteins involved in HN cancer.

The home page presents the database objectives and provide tools for searching genes related to HNSCC, their expression pattern and chromosome location. External data were included in the database to facilitate access to the maximum amount of information on a particular gene or protein. For example, the genes selected by users are linked to PMIDs, metabolic pathways (31, 32, 33, 34), associated ontologies (21), somatic mutations in HN cancer (35), genetic disorders (36) and microarray data. HNSCC microarray data were obtained from GEO (2) and ONCOMINE (3) platforms at the time of manuscript preparation (GEO accession numbers GSE9844, GSE6631, GSE1722, GSE13601, GSE3524, GSE2379, GSE25099 and ONCOMINE dataset Ginos Head-Neck) (37–44) and may help users identify genes with similar expression patterns. Data on proteins, including interactions and drugs that target them (4, 45–53) are also available.

### GO and pathway analysis

The curated set of genes related to HNSCC was imported into DAVID (54, 55), a database for annotation, visualization and integrated discovery (54), and the genes were annotated for GO and pathways using the whole human genome as background. The one-tail Fisher Exact Probability Value was used for gene-enrichment analysis and Bonferroni corrected *P* < 0.05 were considered significant. Ingenuity Pathway Analysis (IPA) software (Qiagen, Redwood City, CA, USA) was also used to identify relevant canonical pathways overrepresented in the set of HNSCC-related genes.

### Database querying

The database is freely available and can be searched at http://www.gencapo.famerp.br/hndb/ with three input forms. By typing the gene symbol, aliases, gene or protein name, accession number or ID into the search box, users can obtain information on whether a gene has already been related to HN cancer. Users can also retrieve all genes related to HN cancer at once and evaluate their expression in HN tumor samples and paired surgical margins, according to eight microarray studies (37–44) selected at the time of the manuscript preparation and described in the ‘Database frameworks and web interface’ section. The search settings are configured to use the official gene symbols, ID numbers and aliases from the National Center for Biotechnology Information (NCBI) or Ensembl Project (56, 57), as well as proteins (by accession number) from the Universal Protein Resource (UniProt) (58).

Users can also browse chromosome regions associated with HN cancer. The data returned by the queries can be downloaded as a spreadsheet or a text file. The results of a particular gene are displayed in a new page that provides the official gene name, gene IDs, aliases, chromosome location and gene expression pattern generated via microarray studies on tumor tissues as well as articles that support its involvement in HNSCC or report prognostic markers. As indicated above, the results also include gene ontologies, metabolic pathways and links to external databases on expression patterns in normal tissues, somatic mutations in cancer and gene-phenotype or disease associations. The protein page provides 3D structures and posttranslational modifications, metabolite and protein–protein interactions, expression patterns and drugs for targets of interest.

## Results and discussion

In total, the ‘Neoplasms by site’ search resulted in 1 819 931 articles (between 2015 and 1928). Two searches for ‘Head and Neck Neoplasms’ resulted in 38 862 and 41 086 articles (between 2015 and 1945), respectively, which after gene2pubmed association and exclusion of redundancy, generated a list of 1611 genes. Following a manual curation, 421 genes not related to HNSCC were excluded and a list of 1190 genes was obtained. To this list, 180 among 517 genes identified by HNOCDB and OrCGDB databases but not detected by our approach were added after a thorough manual reevaluation, resulting in 1370 genes in total. Considering these data, the precision (specificity) of our automated approach was estimated in 74%, and recall (sensitivity) was estimated in 87%. Although these values are satisfactory, they still need to be improved since not all the genes retrieved by the approach were considered relevant after manual curation. In addition, several relevant genes were missed, which indicates that the literature search in future versions of HNdb have to be expanded to include articles identified through digital libraries besides PubMed (e.g. Google Scholar, Web of Science and Scopus) (59–61), and approaches for information extraction should be added, such as NLP based methods.

The analysis of contingency tables constructed using our PMID sets revealed that, although HNSCCs compared to all neoplasms (except HNSCC) show genes with differential citation frequency at the 0.05 level of significance, none of these genes are exclusively associated with HNSCC. In fact, established HNSCC genes listed by (10) (*CCND1*, *CDKN2A*, *EGFR*, *MET*, *PIK3CA*, *PTEN*, *SMAD4*, *TP53*) are also associated with several other tumors (62–69) and all are present in our list of HNSCC-related genes. These results highlight the need of extensive basic and clinical research focused on unique characteristics of this group of carcinomas.

One hundred forty-eight of 1370 genes were linked to at least five PMIDs and thus were classified as top HNSCC-related genes, with *TP53* and *EGFR* being the first two genes of this list (Table 1). These scores for *TP53* and *EGFR* were confirmed by the hypergeometric test (Supplementary Table 1), and indicate that they represent the most extensively studied ones and certainly exhibit relevant results. Regarding the 893 genes mentioned by only one article, many of them probably have not yet been completely exploited as potential markers and deserve further investigations.

**Table 1.** Top HNSCC-related genes

Gene	*N*	Gene	*N*	Gene	*N*	Gene	*N*
TP53	122	NOTCH1	13	ALDH2	7	CD4	5
EGFR	101	VEGFC	13	ANXA2	7	CXCL12	5
CDKN2A	77	MTHFR	12	CASP8	7	CXCL14	5
GSTM1	58	RELA	12	CDK4	7	CYP2D6	5
GSTT1	48	TGFB1	12	CSTA	7	DAPK1	5
CCND1	43	XRCC3	12	CYP1B1	7	ERCC4	5
VEGFA	35	ADH1B	11	FOXM1	7	ERCC5	5
STAT3	34	MIR21	11	H3F3A	7	FASLG	5
MMP9	33	MKI67	11	HPSE	7	FGF2	5
GSTP1	32	TERT	11	ITGB1	7	FN1	5
CD44	31	ADH1C	10	MDM2	7	HGF	5
PTGS2	31	BMI1	10	TIMP3	7	HMGB1	5
XRCC1	31	MLH1	10	XIAP	7	IGF1R	5
BIRC5	28	MMP1	10	CASP3	6	IL10	5
HIF1A	28	PECAM1	10	CD82	6	IVL	5
TP63	28	RB1	10	CDK2	6	JAG1	5
CDH1	26	CDKN1B	9	CDK2AP1	6	KRT19	5
CYP1A1	24	CTTN	9	CXCL8	6	LGALS1	5
MMP2	21	HSP90AB1	9	CXCR4	6	MIR375	5
PTEN	20	MET	9	ENG	6	MTR	5
ERCC2	19	MMP3	9	EPHA2	6	NME1	5
ERCC1	18	SLC2A1	9	FHIT	6	NQO1	5
CDKN1A	17	SOX2	9	KRAS	6	PARK7	5
AKT1	16	TNF	9	KRT14	6	PCNA	5
BCL2	16	AURKA	8	LAMA5	6	PTK2	5
NFKB1	16	DNMT3B	8	MAPK1	6	RHOC	5
BSG	15	EPHX1	8	MSH2	6	SMAD4	5
CCR7	15	FAS	8	MTDH	6	SNAI1	5
ERBB2	15	ITGAV	8	PLAU	6	STAT1	5
HSPA1A	15	MGMT	8	PROM1	6	TGM3	5
OGG1	15	MTOR	8	RAD51	6	TIMP2	5
CYP2E1	14	MYC	8	S100A4	6	TLR4	5
PIK3CA	14	NOS2	8	SKP2	6	TYMS	5
TP73	14	RASSF1	8	SRC	6	VCAN	5
CTNNB1	13	S100A7	8	ABCB1	5	XPC	5
IL6	13	SPP1	8	CA9	5	XRCC5	5
NAT2	13	TWIST1	8	CAV1	5		

*N* = number of PMIDs per gene.

The 1370 HNSCC-related genes showed a heterogeneous distribution along the chromosomes (Table 2) and, as expected, many of them were mapped to known HNSCC ‘hot spots’ such as 11q13 (70, 71). However, several others were mapped to less frequently cited regions. Approximately 10% were mapped to chromosome 1, 7% to chromosome 11 and almost the same amount to chromosome 17, a distribution not correlated with the size in MB of each chromosome.

**Table 2.** Distribution of HNSCC-related genes by chromosome band

Chr	*N*	%	Chr	*N*	%	Chr	*N*	%	Chr	*N*	%	Chr	*N*	%
1p12	2	0.15	3p21-p24	1	0.07	6q16	1	0.07	10p11	5	0.36	17p11	2	0.15
1p21-p13	17	1.19	3p22	4	0.29	6q21	4	0.29	10p13	3	0.22	17p12	1	0.07
1p22	3	0.22	3p24	3	0.22	6q22-q24	6	0.44	10p15-p14	5	0.36	17p13	14	0.98
1p32-p31	8	0.58	3p25	7	0.51	6q25	5	0.36	10q11	5	0.36	17q	1	0.07
1p36-p34	38	2.66	3p26	2	0.15	6q26	2	0.15	10q21	4	0.29	17q11	13	0.91
1q21	23	1.61	3q12-q13	7	0.51	7p11	2	0.15	10q22	5	0.36	17q12	5	0.36
1q21-q22	1	0.07	3q21	3	0.22	7p12	3	0.22	10q23-26	36	2.52	17q12-q21	1	0.07
1q21-q25	1	0.07	3q22	1	0.07	7p13	1	0.07	11p12-p11	4	0.29	17q21	25	1.75
1q22-q23	4	0.29	3q23	1	0.07	7p14	3	0.22	11p13	3	0.22	17q21-q22	2	0.15
1q23	9	0.66	3q25-q27	9	0.66	7p15-p13	5	0.36	11p15-p14	31	2.17	17q22	2	0.15
1q24	1	0.07	3q27	4	0.29	7p21	5	0.36	11q11	1	0.07	17q22-q23	1	0.07
1q25	5	0.36	3q28-q29	4	0.29	7p22	6	0.44	11q12	4	0.29	17q23	5	0.36
1q25-q31	2	0.15	4p14-p13	1	0.07	7q11	5	0.36	11q13	30	2.1	17q23-q25	1	0.07
1q31-q32	6	0.44	4p15	1	0.07	7q21	8	0.58	11q14-q21	5	0.36	17q24	5	0.36
1q32	12	0.88	4p16	2	0.15	7q21-q22	3	0.22	11q22-q23	23	1.61	17q25	13	0.91
1q32-q41	1	0.07	4q11-q12	1	0.07	7q22	9	0.66	11q24	2	0.15	18p11	3	0.22
1q41-q42	11	0.77	4q12	4	0.29	7q31	9	0.66	12p13-p12	27	1.89	18q11	3	0.22
1q43	1	0.07	4q12-q13	1	0.07	7q32	5	0.36	12q11-q13	23	1.61	18q12	3	0.22
1q43-1q23	1	0.07	4q13	9	0.66	7q32-q34	1	0.07	12q14-q15	9	0.66	18q21	15	1.09
1q44	2	0.15	4q13-q21	1	0.07	7q34	3	0.22	12q21	5	0.36	18q22	1	0.07
2p12	3	0.22	4q21	7	0.51	7q34-q35	1	0.07	12q22	1	0.07	18q23	1	0.07
2p12-p11	1	0.07	4q22	3	0.22	7q35-q36	1	0.07	12q23	1	0.07	19p12	1	0.07
2p13	3	0.22	4q23-q25	10	0.70	7q36	3	0.22	12q24	15	1.09	19p13	24	1.68
2p13-p12	1	0.07	4q26	2	0.15	8p12-p11	6	0.44	13q11-q13	8	0.58	19q12	1	0.07
2p14	1	0.07	4q27	2	0.15	8p21	8	0.58	13q14	5	0.36	19q13	39	2.73
2p21	3	0.22	4q28	1	0.07	8p21-p12	1	0.07	13q21	1	0.07	20p12-p11	3	0.22
2p21-p16	1	0.07	4q31	6	0.44	8p22	6	0.44	13q22	3	0.22	20p13	3	0.22
2p22	3	0.22	4q32	2	0.15	8p22-p21	1	0.07	13q31	1	0.07	20pter-p12	1	0.07
2p24	2	0.15	4q34-q35	6	0.44	8p23	8	0.58	13q33-q34	4	0.29	20q11	8	0.58
2p25	2	0.15	5p13	5	0.36	8q11	4	0.29	14q11-q12	9	0.66	20q12-q13	8	0.58
2q11	2	0.15	5p15	5	0.36	8q12	1	0.07	14q13	3	0.22	20q13	12	0.88
2q12	1	0.07	5q11	2	0.15	8q13	3	0.22	14q22-q23	6	0.44	21q21	2	0.15
2q13	1	0.07	5q12-q13	2	0.15	8q21	5	0.36	14q24	5	0.36	21q22	9	0.66
2q14	2	0.15	5q13	2	0.15	8q22	4	0.29	14q31-q32	20	1.4	22q11	8	0.58
2q21	3	0.22	5q14	4	0.29	8q22-q23	1	0.07	15q11	1	0.07	22q12	3	0.22
2q22	1	0.07	5q21-q22	1	0.07	8q23	5	0.36	15q12	1	0.07	22q13	18	1.26
2q23-q24	3	0.22	5q23	2	0.15	8q24	16	1.12	15q13	2	0.15	Xp11	5	0.36
2q31	3	0.22	5q31	12	0.88	9p12	1	0.07	15q14	3	0.22	Xp21	3	0.22
2q31-q32	1	0.07	5q31-q32	1	0.07	9p13	4	0.29	15q15-q21	5	0.36	Xp22	7	0.51
2q32	2	0.15	5q32	5	0.36	9p21	5	0.36	15q22	7	0.51	Xp22-p21	1	0.07
2q33	4	0.29	5q33	2	0.15	9p22	3	0.22	15q23-q24	9	0.66	Xq11	1	0.07
2q33-q34	3	0.22	5q34	3	0.22	9p23-p24	1	0.07	15q25-q26	7	0.51	Xq12	2	0.15
2q34	2	0.15	5q35	8	0.58	9p24	3	0.22	16p11	3	0.22	Xq13	1	0.07
2q35	9	0.66	6p12	6	0.44	9q21	6	0.44	16p12	4	0.29	Xq21-q22	4	0.29
2q35-q37	1	0.07	6p21	28	1.96	9q22	8	0.58	16p13	12	0.88	Xq23	1	0.07
2q36	2	0.15	6p22	2	0.15	9q31	5	0.36	16q12	1	0.07	Xq25	1	0.07
2q37	16	1.12	6p23	1	0.07	9q32	2	0.15	16q13	4	0.29	Xq26	5	0.36
3p11	1	0.07	6p24	4	0.29	9q32-q33.3	2	0.15	16q21-q22	12	0.88	Xq28	9	0.66
3p14	2	0.15	6p25	2	0.15	9q33	7	0.51	16q23	1	0.07	ND	4	0.29
3p21	17	1.19	6q14	2	0.15	9q34	15	1.09	16q24	4	0.29			

Chr = chromosome band; *N* (%) = number of genes and percentage; ND = not determined.

To evaluate the performance of our literature mining approach, we compared our nonredundant list of 1190 genes with the top genes selected in HNOCDB and OrCGDB (currently frozen) databases. Considering the same anatomical sites analyzed in the present work, HNOCDB extracted 133 genes in oral, 14 in tongue, 7 in hypopharyngeal, 3 in oropharyngeal and 60 in laryngeal cancers through text-mining. OrCGDB selected 374 genes involved in oral cancer by searching PubMed abstracts and MeSH terms. A total of 517 nonredundant genes was identified by these databases. After a manual curation, 180 genes retrieved from HNOCDB and OrCGDB were added to our list of 1190 genes. In contrast with these databases, the present study performed three searches using MeSH terms and was more stringent by excluding articles that also analyzed non-HNSCC tumors. Therefore, our gene list (the largest of the three databases) is more specific and, therefore, more focused on the tumors of interest. In addition, the IPA showed that the top canonical pathway associated with our 1190 genes is the Molecular Mechanisms of Cancer (*P* = 6.64^−^^66^, overlap 34.5%, 126/365), thus supporting their relevance in the neoplastic process. Differently, this pathway was not associated with OrCGDB and HNOCDB genes (*n* = 517), which showed as the top-ranked pathways Aryl Hydrocarbon Receptor Signaling (*P* = 1.42E^−^^32^, overlap 29.3% 41/140), Bladder Cancer Signaling (p-value 4.44E^−^^32^, overlap 39.1% 34/87) and Hepatic Fibrosis/Hepatic Stellate Cell Activation (*P* = 5.49E^−^^32^, overlap 24.6% 45/183). Furthermore, HNdb is the only database that uses specific MeSH terms to link genes to literature data on prognosis and outcome (Supplementary Table 2, also available on the gene results page), facilitating the identification of markers that are relevant to tumor biology and therapy response.

**Figure 2.**
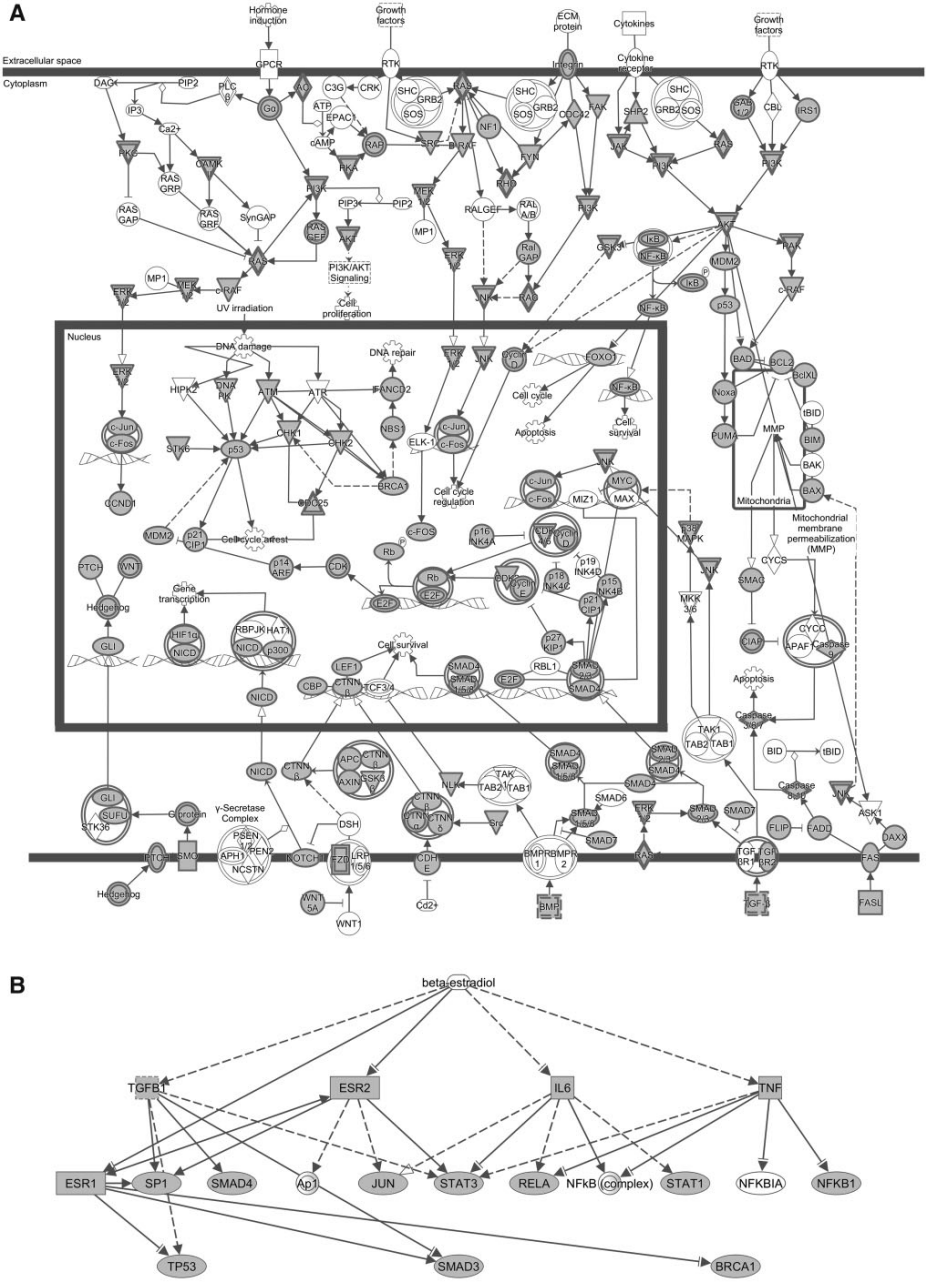
Biological networks according to IPA of 1370 genes related to HNSCC. (A) The canonical pathway ‘Molecular mechanisms of cancer’ and the growing picture of molecular mechanisms in HNSCC: receptor signaling pathways are induced by hormone, growth factors, extracellular matrix proteins or cytokines, leading to the activation of different cytoplasmatic effectors and transcription factors, and promoting cell cycle regulation, DNA repair, apoptosis, and cell survival and proliferation. (B) Beta-estradiol as an upstream regulator in head and neck tumorigenesis: network enriched in transcription factors/regulators (*ESR1* and *2*, *JUN, TP53*, *NFKB1*, *RELA*, *STAT1* and *3*, *SP1*), signal transducers (*SMAD3* and *4*), DNA repair protein (*BRCA1*) and proinflammatory cytokines (*IL6*, *TGFB1*, *TNF*) identified by the present study. Solid arrows = known interactions; dotted arrows = indirect interactions; inverted triangle = kinase; double circle = complex; circle, ellipse, diamond or square = other; filled shapes = genes from our list; nonfilled shapes = genes that are part of the network but not part of the list.

To investigate the biological meaning of the HNSCC-related genes, we performed a GO and pathway analyses using DAVID tools. A total of 1329 DAVID identifiers were mapped from the list of 1370 genes and similar annotation terms were clustered into groups, removing redundancy. More than 500 of annotation clusters were obtained, 86 of them with enrichment scores >5.0 and Bonferroni corrected *P* < 0.05 (Supplementary Table 3). The results showed an overrepresentation of clusters related to tissue development and differentiation, response to stimulus, signal transduction, cell proliferation, cell migration, apoptosis, transcription and cell adhesion, which are biological processes relevant to cancer. In addition, the top five canonical pathways identified by the IPA for these 1370 genes were Molecular Mechanisms of Cancer (Figure 2A), Colorectal Cancer Metastasis Signaling, Role of Macrophages, Fibroblasts and Endothelial Cells in Rheumatoid Arthritis, Pancreatic Adenocarcinoma Signaling and IL-8 Signaling (*P* = 4.90E ^−^ ^71^, 1.95E ^−^ ^58^, 7.11E ^−^ ^56^, 5.46E ^−^ ^53^, 2.25E ^−^ ^48^, respectively), thus strongly validating our strategy and the informative characteristic of the set of genes.

Furthermore, the diversity of results compiled in our dataset allowed identifying novel and mostly unexplored gene associations. For example, the DAVID analysis revealed that processes related to response to steroid hormone stimulus were significantly enriched in our list of genes (enrichment scores = 37.99, Bonferroni correction, *P*_corr_ = 7.50E ^−^ ^31^) and IPA showed beta-estradiol as one of the top upstream regulators (*P*-value of overlap = 3.35E ^−^ ^163^), ranking next to *TGFB1*, *TNF* and *TP53* (Figure 2B). Few studies have explored the metabolic pathways involved in the response to steroid stimulus in HNSCC. Egloff and collaborators (72) observed that estrogen induces activation of members of the mitogen- activated protein kinase (MAPK) family in HNSCC cell lines. The authors also reported evidence that estrogen receptor and epidermal growth factor receptor cross talk is present in HNSCC. In turn, Brooks and collaborators (73) found that increased levels of estrogen receptor β promotes *NOTCH1* expression and differentiation of HNSCC cells both *in vitro* and *in vivo*. Thus, we demonstrate that a database integrating multiple types of data greatly expands the possibilities for gene networks investigation, providing potential associations to be tested.

## Conclusions

Despite the development of tools to mine vast amounts of genomic data, to our knowledge, there is no initiative to curate and compile information from literature regarding genes, proteins, metabolic pathways, diseases, prognosis/outcomes and drugs associated with HNSCC. The HNdb is an effort toward this goal and is intended to be an integrated database with rapid and easy-to-use tools that facilitate literature and biological data mining to thereby promote research and generate new insight into the development of useful markers for HN cancer.

## Supplementary data

Supplementary data are available at *Database* Online.

Supplementary Data
