# Supplementary material for: HNdb: an integrated database of gene and protein information on head and neck squamous cell carcinoma
Source: Database (Oxford). 2016 Mar 24;2016:baw026. doi: 10.1093/database/baw026 (PMC4806539; doi:10.1093/database/baw026)
Supplement: Supplementary Data [file supp_2016_baw026_index.html]

Supplementary Data 

# HNdb: an integrated database of gene and protein information on head and neck squamous cell carcinoma

## Supplementary Data

files

- Supplementary Data - zip file
