# Supplementary material for: HNdb: an integrated database of gene and protein information on head and neck squamous cell carcinoma
Source: Database (Oxford). 2016 Mar 24;2016:baw026. doi: 10.1093/database/baw026 (PMC4806539; doi:10.1093/database/baw026)
Supplement: Supplementary Data [file supp_baw026_suppl_data.zip › Supplementary File 1.doc]

**Supplementary File 1. Systematic search strategy used to identify studies on HNSCC.**

**Literature search strategy**

A literature review was conducted in the PubMed database to identify studies and genes related with HNSCC. The methodological strategy combined two initial automated stages (I and II) and a separate manual step.

In stage I, the studies were retrieved from PubMed database using a combination of Medical Subject Headings (MeSH) terms and Boolean operators. Three literature searches were performed using no filters. The first search (A) included the broad MeSH term “Head and Neck Neoplasms” and excluded terms of the MeSH hierarchy that do not meet criteria for squamous cell carcinomas at the anatomical sites considered in this study (gingiva, palate, tongue, oropharynx, hypopharynx and larynx). The second search (B) included only the MeSH terms that met the above criteria. Finally, the third search (C) employed the term “Neoplasms by site”[MH] and the results obtained were only used in contingency tables for comparative purposes. The tag [MH] retrieves any article indexed with a MeSH term (not necessarily a major topic) (Box 1).

In searches A and B, the MeSH terms were followed by the syntax [MH:noexp] to turn off the automatic inclusion of more specific terms. Both searches were performed to ensure the correct retrieval of most studies on the subsites of interest. Since each PubMed article record may include several MeSH terms through which the manuscript can be recovered (both MeSH Major Topic as well as MeSH Subheadings), their results are never identical but may complement each other. For example, the article PubMed Identifier (PMID) 23800673 was indexed by PubMed with 21 MeSH terms, including "gingival neoplasms" and "thyroid neoplasms". Our search B retrieved this record because it contains the "gingival neoplasms" MeSH term and this search asked for all records with this term. However, search A did not retrieve this citation because we chose not to include any records linked to "thyroid neoplasms" term. The data from searches A and B were combined and repeated records were excluded.

In stage II, the articles selected in stage I were associated with genes using the gene2pubmed association file (<ftp://ftp.ncbi.nih.gov/gene/DATA/gene2pubmed.gz>), which contains the Gene Identifiers (Gene IDs) and the respective PMIDs. For this association, only genes from humans were considered. The PMIDs thereby obtained were downloaded via PubMed and compiled. Publications assigned to MeSH terms for head and neck neoplasms were manually curated by two independent investigators, and those with no direct involvement with HNSCC were excluded. Likewise, studies that analyzed other tumor types in addition to HNSCC were excluded since they might introduce non-specific data.

Genes/PMIDs not detected by our approach but selected by two other databases also focusing on head and neck cancer (HNOCDB - http://gyanxet.com/hno.html and OrCGDB - http://www.tumor-gene.org/Oral/oral.html) were included in our list of genes after manual curation.

Due to the importance of identifying prognostic signatures for HNSCC as well as markers associated with disease progression, an independent search was performed using MeSH and non-MeSH terms related to “Metastasis” and “Prognosis/Outcome” against abstracts and titles of the manually curated PMID set of articles (Box 1).

**Box 1. Search terms used for the identification of studies in the PubMed database.**

Search A included the broad MeSH term “Head and Neck Neoplasms” followed by the Boolean operator NOT before each term of the MeSH hierarchy that did not meet criteria for squamous cell carcinomas at the anatomical sites considered in this study. Search B included only terms of interest followed by the Boolean operator OR. Search C employed the term “Neoplasms by site”. The “Metastasis” and “Prognosis/Outcome” searches included MeSH terms of interest and also non-MeSH terms*, as well as the Boolean operator OR.

| **Search type** | **MeSH terms** | **Search type** | **MeSH terms** |
| --- | --- | --- | --- |
| **A** | Head and Neck Neoplasms | **Metastasis** | Lymphatic Metastasis |
|  | Ear Neoplasms |  | Neoplasm Invasiveness |
|  | Esophageal Neoplasms |  | Neoplasm Metastasis |
|  | Facial Neoplasms |  | Neoplasm Micrometastasis |
|  | Leukoplakia, oral |  | Neoplasm Seeding |
|  | Lip Neoplasms |  | Neoplastic Cells, Circulation |
|  | Nasopharyngeal Neoplasms |  | Treatment Outcome |
|  | Nose Neoplasms |  |  |
|  | Parathyroid Neoplasms | **Prognosis/Outcome** | Disease Progression |
|  | Salivary Gland Neoplasms |  | Disease-free Survival |
|  | Thyroid Neoplasms |  | Fatal Outcome |
|  | Tracheal Neoplasms |  | Follow-up Studies |
| **B** | Gingival Neoplasms |  | Neoplasm Grading |
|  | Palatal Neoplasms |  | Neoplasm Staging |
|  | Tongue Neoplasms |  | Nomogram |
|  | Oropharyngeal Neoplasms |  | Prognosis |
|  | Hypopharyngeal Neoplasms |  | Survival Rate |
|  | Laryngeal Neoplasms |  | Treatment Failure |
| **C** | Neoplasms by site |  | Treatment Outcome |

*Non-MeSH terms: histological grading, lymphatic invasion, organ invasion, perineural invasion, peritumoral infiltration, peritumoral inflammation, vascular invasion.

**USE CASE**

**Searching information on genes/proteins involved in Head and Necks Squamous Cell Carcinoma** (also in Supplementary Figure 1).

1. Go to HNdb home page (<http://www.gencapo.famerp.br/hndb/>)
2. From the links on the Search box, select **All HNSCC related genes** for all HNSCC related genes compiled by the database, OR select **HNSCC related chromosome regions** for chromosome regions where HNSCC related genes have been mapped, OR select **By gene** and write your gene of interest OR select **By gene list** and include several genes of interest.
3. Press **Enter** or **Search** to complete this step
4. At the **All HNSCC related genes** or **HNSCC related chromosome regions** pages, you may download a TXT file containing the data by clicking the corresponding buttons on the right.
5. At the **Concise Results** page on your gene(s) of interest, you can see official symbol(s) and name(s), gene identifier(s) and score(s) for gene-to-HNSCC association determined by a hypergeometric test (see references in Henrique et al, Database). Next, select your gene and press **Enter**. The **Full Results** page will be displayed.
6. Under the **Full Results** page, a list of data is available, including official gene symbol and name, aliases, chromosome location, gene type, gene ontologies, gene-related diseases, somatic mutations, and protein subcellular location. You may download a PDF file or a TXT file containing the data by clicking the corresponding buttons on the right. External links are also available to facilitate access to the maximum amount of information on a particular gene or protein:

- gene/protein identifiers from Entrez gene [http://www.ncbi.nlm.nih.gov](http://www.ncbi.nlm.nih.gov/)
- PMIDs related to HNC from PubMed <http://www.ncbi.nlm.nih.gov/pubmed>
- KEGG pathways from KEGG <http://www.genome.jp/kegg/pathway.html>
- Reactome pathways from Reactome [http://www.reactome.org](http://www.reactome.org/)
- microarray data and expression pattern from GEO <http://www.ncbi.nlm.nih.gov/geo> and Oncomine <https://www.oncomine.org/resource/login.html>
- protein 3-D structure from Protein Data Bank <http://www.rcsb.org/pdb/home/home.do>
- drugs to target proteins from DrugBank <http://www.drugbank.ca/>
- protein interactions from BioGRID <http://thebiogrid.org/>
- PMIDs related to HNC prognosis/outcome from <http://www.ncbi.nlm.nih.gov/pubmed>

7. An “on line help” (?) is available throughout the site to help the user understand each step of the workflow.
